# End-to-End Multimodal Multiple Instance Learning for Cancer Histopathology Classification with Dual-Attention Fusion

**DOI:** 10.1007/s10916-026-02379-0

**Published:** 2026-04-15

**Authors:** Satoshi Shirae, Shyam S. Debsarkar, Hiroharu Kawanaka, Bruce J. Aronow, V. B. Surya Prasath

**Affiliations:** 1 Graduate School of Engineering, Mie University, 1577 Kurima-machiya, Tsu, 514-8507 Mie Japan; 2 Department of Computer Science, University of Cincinnati, 2600 Clifton Ave, Cincinnati, OH 45221 USA; 3 Division of Biomedical Informatics, Cincinnati Children’s Hospital Medical Center, 3333 Burnet Ave, Cincinnati, OH 45229 USA

**Keywords:** Deep learning, CNN, Multiple instance learning, Histopathology, Whole slide images, Gene expression profiles, Glioma, Lung cancer, Breast cancer, Multimodal

## Abstract

In recent years, computational pathology has advanced toward integrating histopathological images and genomic data to improve diagnostic accuracy and biological interpretability. In this study, we propose a lightweight end-to-end multi-instance learning model that integrates whole slide images (WSIs) with gene expression profiles. The proposed method efficiently extracts image features using a reduced MobileNetV4 and treats gene information at the gene set level based on Gene Set Enrichment Analysis (GSEA) to capture functional relationships among genes. Image and gene features are aggregated within and across modalities through self-attention and cross-attention mechanisms. Our experiments on three cancer types, namely low-grade glioma (LGG), non-small cell lung cancer (NSCLC), and breast cancer (BRCA) showed that the proposed model outperformed image-only models and conventional single-vector gene input models. In particular, the ROC-AUC reached 0.740 for LGG, 0.982 for NSCLC, and 0.966 for BRCA, with a notable improvement in PR-AUC observed for BRCA classification. These results indicate that the integration of morphological and molecular information is effective for capturing disease characteristics. Furthermore, the proposed model maintains high classification performance while reducing computational resources, suggesting its potential applicability to large-scale pathological datasets and clinical applications.

**Supplementary Information:**

The online version contains supplementary material available at 10.1007/s10916-026-02379-0.

## Introduction

In modern clinical practice, histopathological diagnosis remains the gold standard for diagnosing many cancers [1]. With the advancement of whole-slide imaging (WSI) technology, it has become possible to digitize entire glass slides, enabling initial pathological diagnoses, intraoperative frozen section consultations [2, 3], and remote expert review without incurring the costs or delays associated with physical slide transportation [4]. However, most pathological diagnoses still rely heavily on pathologists’ subjective observations, leading to an increasing demand for quantitative analysis of WSIs [5]. To address this challenge, automated image analysis using artificial intelligence (AI) has recently attracted significant attention. The application of AI is expected to improve diagnostic efficiency, reproducibility, and accuracy [6].

In recent years, convolutional neural networks (CNNs) have been widely applied to image classification tasks using AI. Nevertheless, whole slide images (WSIs) are extremely high-resolution and contain massive amounts of data, making it infeasible to analyze an entire slide directly. Consequently, WSIs are typically divided into tiled patch images for analysis. Because some patches contain tumor regions while others do not, labeling each patch individually would be required, however this is highly labor-intensive and impractical for pathologists. Therefore, analyses must rely solely on slide-level labels. Under these circumstances, multiple instance learning (MIL) has become a key approach for WSI analysis [7]. MIL is a type of weakly supervised learning that predicts the label of a “bag” composed of multiple “instances.” In the context of WSI analysis, patch images correspond to instances, and the entire slide corresponds to a bag. Typically, MIL-based analysis of WSIs consists of three main stages: feature extraction from patches, aggregation of patch-level information, and final prediction. Since extracting complex pathological features requires a powerful representation model, deep learning model pre-trained on large datasets are often employed. However, such deep models demand substantial computational resources, and many prior studies have separated the feature extractor from the classifier [8, 9], resulting in non-end-to-end MIL models [10–12]. In contrast, end-to-end learning is crucial for extracting image features optimized for the classification task. To address this, we previously proposed an MIL model that employs a reduced MobileNetV4 [13] as a lightweight feature extractor. Our lightweight end-to-end MIL model successfully reduced computational resources while maintaining classification performance in glioma subtype classification [14].

Despite the growing attention toward MIL-based WSI analysis, the integration of multiple data modalities remains limited. In clinical settings, clinicians typically evaluate information from multiple modalities such as imaging, molecular profiling, and clinical data to guide diagnosis, prognosis, and treatment strategy [15]. Therefore, incorporating multimodal data in AI-based analysis is essential. Several studies have focused on molecular genetic analysis, demonstrating that tumor genotypes can complement histopathological diagnosis [16]. In particular, gene expression profiling has the potential to provide an objective means for tumor classification [17, 18]. Based on this rationale, this study addresses cancer classification tasks that integrate both histopathological WSIs and RNA-seq-derived gene expression profiles. We specifically focus on three classification settings: glioma grade (Grade II vs. Grade III), non-small-cell lung cancer (LUAD vs. LUSC), and breast cancer molecular subtype (Luminal A vs. TNBC).

Glioma is the most common tumor arising in the central nervous system, and accounts for approximately 80% of malignant primary brain tumors [19–21]. Diffuse gliomas are classified by the World Health Organization (WHO) into grades II to IV based on histopathological and molecular genetic characteristics [22, 23]. Among them, diffuse gliomas of grades II and III are defined as low-grade gliomas (LGG). Treatment strategies for LGG differ depending on tumor grade: postoperative LGG III patients generally receive adjuvant therapies such as radiotherapy or chemotherapy, whereas younger postoperative LGG II patients may be monitored by imaging follow-up such as MRI alone. Therefore, the accurate diagnosis of malignancy is required [24–30]. Truong et al. [31] developed a deep learning model using whole slide images (WSIs) for glioma grading. They demonstrated that distinguishing between grades within LGG remains challenging even with deep learning models and concluded that capturing intratumoral heterogeneity between grades is a major issue. Pei et al. [32] proposed a deep neural network (DNN) model that integrates pathological image features and molecular marker features for glioma grading. Their study demonstrated that incorporating molecular information consistently improved classification performance compared to using pathological features alone, thereby highlighting the importance of multimodal analysis. Lung cancer is one of the most prevalent and fatal cancers worldwide, with high mortality rates in both men and women [33]. Lung cancer is broadly classified into small-cell lung cancer (SCLC) and non-small-cell lung cancer (NSCLC). NSCLC accounts for approximately 85-90% of all lung cancers and mainly comprises two subtypes: lung squamous cell carcinoma (LUSC) and lung adenocarcinoma (LUAD) [34]. Generally, LUAD progresses more slowly than LUSC at the same stage but tends to metastasize earlier [35]. Moreover, the molecular mechanisms of tumorigenesis differ substantially between LUAD and LUSC, and treatments effective for LUAD often fail to show efficacy in LUSC [36]. Coudray et al. [37] developed a deep learning model based on WSIs for NSCLC subtype classification and mutation prediction. They concluded that subtype classification using a deep learning model (InceptionV3) can assist in lung cancer diagnosis. Perez-Carillo et al. [38] investigated NSCLC classification using a late fusion model that integrates WSIs and RNA-Seq data. They demonstrated that the fusion model outperformed models using single data sources and suggesting that such multimodal models may improve patient care. Breast cancer is the most common malignancy among women, and breast invasive carcinoma (BRCA) is known as a highly heterogeneous disease [39, 40]. It consists of several biologically distinct subtypes, and understanding these subtypes enables the selection of optimal treatments for individual patients [41]. Breast cancers are classified into five subtypes, namely Luminal A, Luminal B, HER2-positive, triple-negative breast cancer (TNBC), and unclear based on immunohistochemical markers for ER, PR, and HER2 [42]. Luminal A is the most prevalent subtype and is associated with the most favorable prognosis among them [43, 44]. In contrast, TNBC is characterized by the absence of ER, PR, and HER2 expression, exhibits rapid progression and metastasis, and is associated with poor prognosis due to the lack of available targeted therapies. Lin et al. [45] developed a deep neural network for breast cancer subtype classification using multi-omics data. They demonstrated that integrating multiple omics modalities achieved higher accuracy than using single-omics data alone, thereby improving the recognition of breast cancer subtypes.

Based on these backgrounds, we propose a lightweight end-to-end multimodal MIL model that integrates histopathological WSIs and gene expression profiles. The proposed method aims to enhance both classification performance and interpretability by jointly analyzing morphological information extracted from WSIs and molecular information represented at the gene set level. We validated the effectiveness of the proposed model on LGG, NSCLC and BRCA, and evaluated the advantages of multimodal analysis through comparisons with single-modality approaches and conventional methods.

**Fig. 1:**
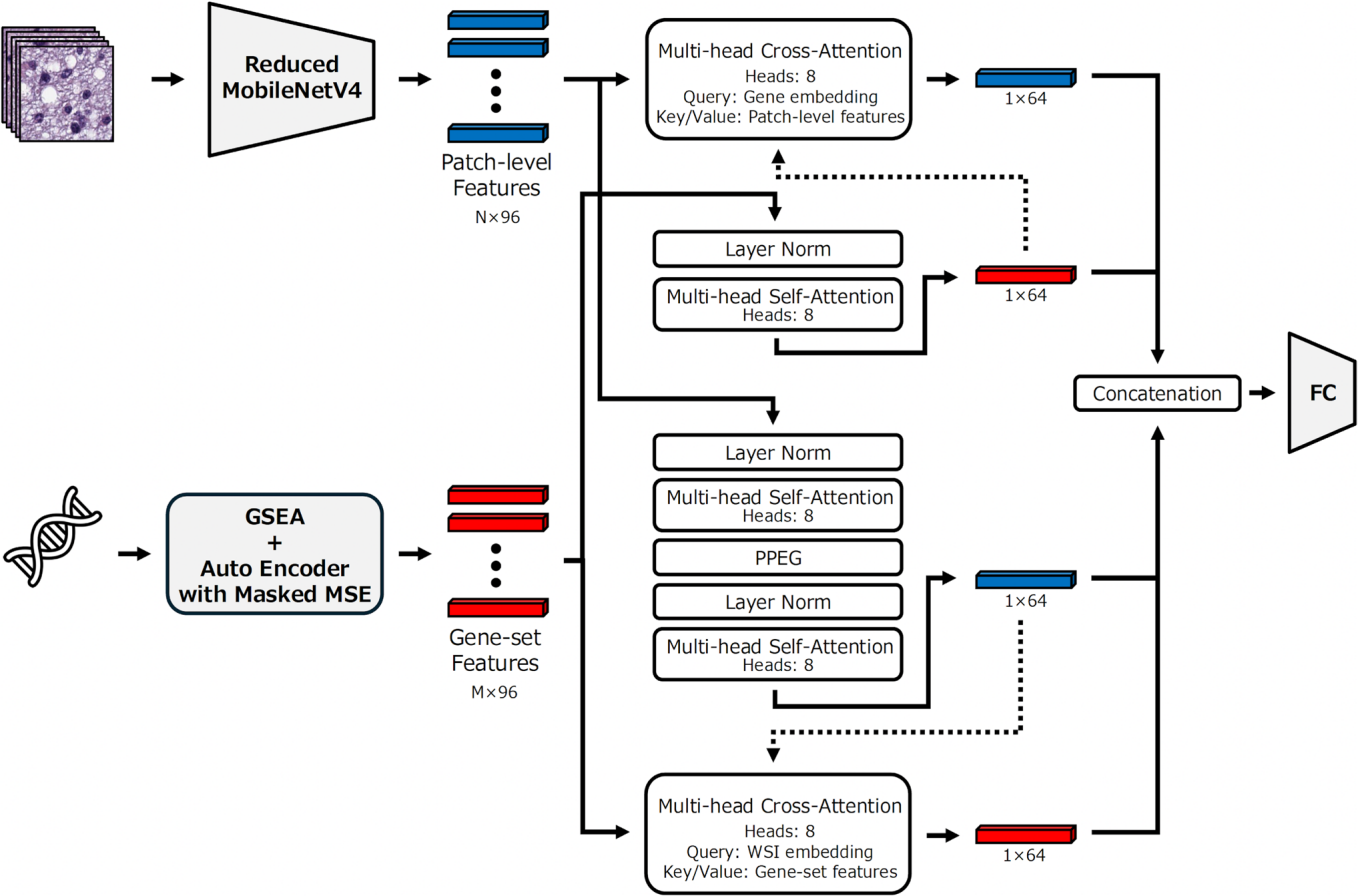
Overview of the proposed end-to-end multimodal MIL framework. For histopathological images, a reduced MobileNetV4 is used for efficient feature extraction. Gene features are handled at the gene set level, enabling the model to learn functional relationships among genes. Image and gene features are respectively aggregated into a single vector using a self-attention mechanism. Furthermore, cross-attention is applied in two directions: one uses pre-aggregated image features as keys and values and the aggregated gene feature vector as the query, while the other uses pre-aggregated gene features as keys and values and the aggregated image feature vector as the query. As a result, each modality obtains two aggregated vectors. All aggregated vectors are concatenated and used for classification

## Materials

### Proposed Method

An overview of the proposed method is shown in Fig. 1. The proposed method is a lightweight end-to-end MIL model that takes histopathological images and gene expression profiles as inputs. In conventional MIL-based histopathological image analysis, deep learning models such as ResNet50d have typically been used for patch-level feature extraction. However, such feature extractors are separated from the classifier due to their high computational resources. As a result, image features optimized for the classification task cannot be learned in an end-to-end manner. To address this limitation, we propose an end-to-end MIL model in which the feature extractor and classifier are jointly optimized.

From the perspective of training stability and convergence, it is desirable to use a feature extractor that is pre-trained on large-scale datasets, such as ImageNet. In this study, we adopt MobileNetV4 as the feature extractor, which is known to be both lightweight and highly performant among pretrained models. Additionally, we focus on the Universal Inverted Bottleneck (UIB) blocks present in MobileNetV4. UIBs are designed to improve efficiency and generalization performance in complex tasks such as multiclass classification. However, retaining all UIBs is considered redundant for the binary classification task addressed in this study. Accordingly, ten removable UIBs were eliminated to improve computational efficiency. Furthermore, channel redundancy was evaluated using the Average Percentage of Zero (APoZ) [46] for the high-channel convolutional layers in the final stage of the model. APoZ measures the proportion of activations that become zero after the activation function, and channels with high APoZ values are known to contribute less to feature extraction. Therefore, redundant channels were removed based on APoZ to further improve computational efficiency.

For aggregating patch-level image features, a Transformer Encoder based on TransMIL was employed [11]. This module consists of Layer Normalization, Multi-head Self-Attention, and a Pyramid Position Encoding Generator (PPEG). Through PPEG, spatial information is preserved during feature aggregation, enabling the extraction of a one-dimensional vector representation of the WSI. TransMIL adopts an approximate Transformer based on Nyström Attention to reduce the computational cost of self-attention, and the same configuration was used in this study [47].

In conventional gene expression analysis, the expression levels of individual genes are typically converted into a one-dimensional vector and input into a model as independent features [14]. However, such representations make it difficult to adequately capture functional relationships among genes and structural information based on biological pathways. To address this issue, we adopt gene set-level representations based on Gene Set Enrichment Analysis (GSEA) [48]. Each gene set consists of groups of genes defined according to existing biological knowledge. Since this gene set features do not possess a spatial structure, they were aggregated using a Transformer Encoder that does not include PPEG. Nyström Attention was also applied in this aggregation process to reduce the computational complexity of self-attention. This process does not directly model interactions between gene sets. Instead, gene sets are aggregated by considering their relative contributions to classification through attention weights.

Furthermore, patch-level image features and gene set features are aggregated using a cross-attention mechanism. In each cross-attention module, the image features and gene features aggregated by self-attention are used as queries. This cross-attention mechanism aims to capture the relevance for classification between morphological information derived from histopathological images and molecular biological information based on gene expression by cross-referencing the two modalities. Through these processes, a total of four one-dimensional feature vectors were derived from self-attention and cross-attention mechanisms. Finally, these feature vectors are concatenated and input into the classification module, enabling integrative classification that jointly exploits information from both pathological images and gene expression profiles.

In the proposed method, end-to-end learning refers to a training paradigm in which all learnable parameters including patch-level image feature extraction, attention-based aggregation modules, and the final classification layer are jointly optimized, except for the identification of gene sets based on GSEA and the extraction of latent features using an autoencoder. This end-to-end optimization enables the learning of feature representations that are specifically optimized for the classification task.

**Fig. 2:**
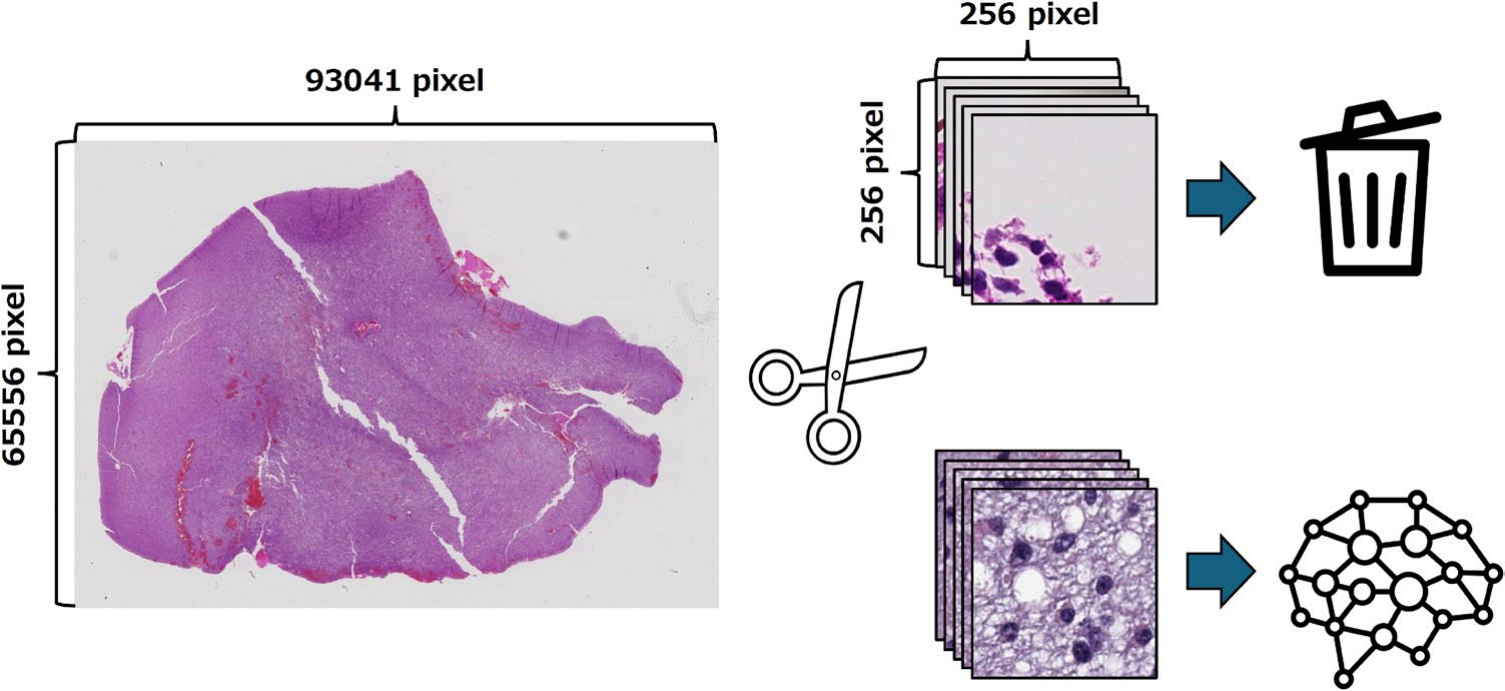
Preprocessing workflow of WSIs. Since giga pixel-level WSIs are difficult to process directly, they were divided into 256 $\times$ 256 patch images. Patch images that do not sufficiently contain tumor regions and have large background areas were excluded from the analysis

**Table 1:** Number of significant gene sets identified by GSEA. This table shows the number of significant gene sets when GO- and KEGG-based GSEA were applied as a preprocessing step to the gene expression profiles. Significant gene sets were determined using an adjusted p-value threshold of < 0.01

Task	Number of significant GO terms	Number of significant KEGG pathways
LGG II vs. LGG III	950	84
LUAD vs. LUSC	154	12
Luminal A vs. TNBC	77	3

**Fig. 3:**
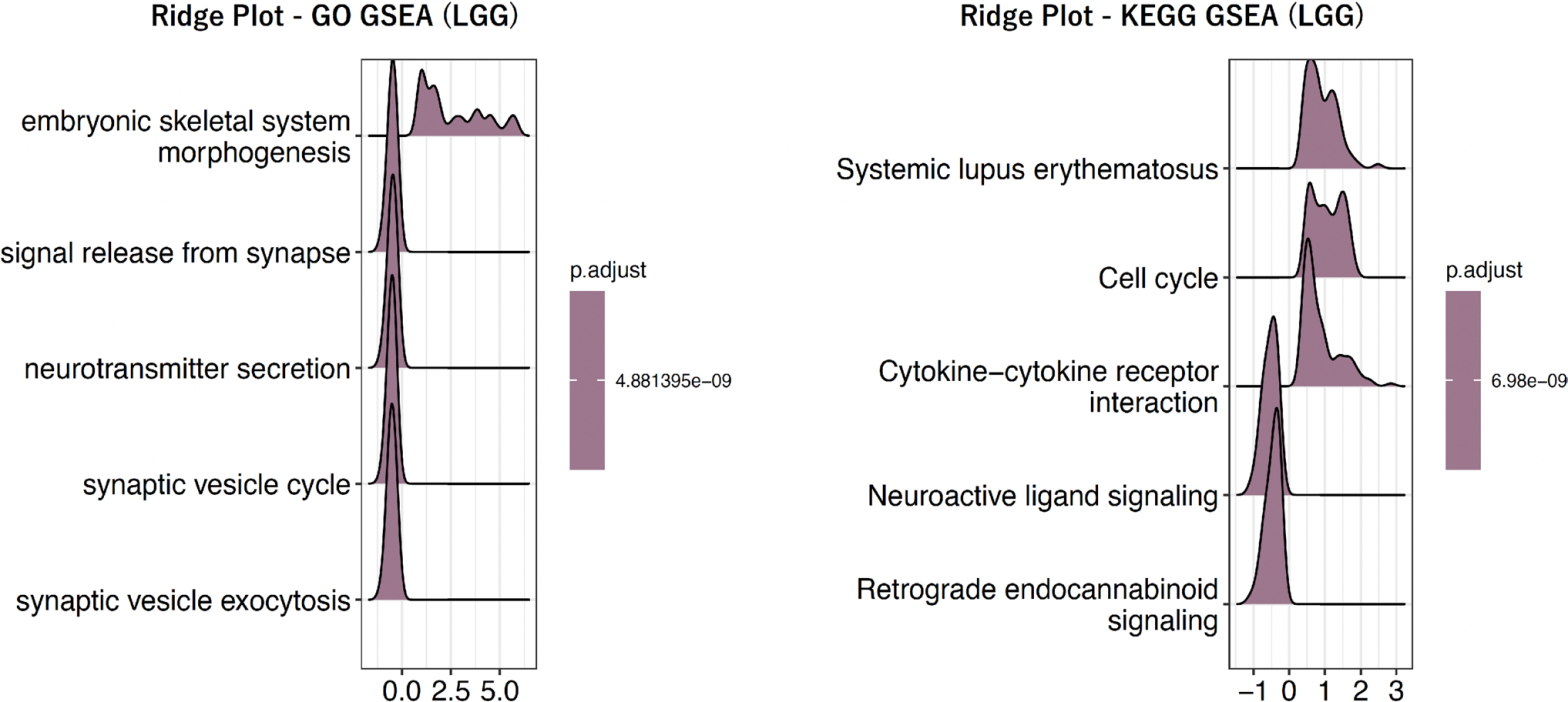
Ridge plot of GSEA for LGG classification. Ridge plots of the top gene sets that were most significantly enriched in GO- and KEGG-based GSEA for LGG classification. The left panel shows the results of GO-based GSEA, while the right panel shows those of KEGG-based GSEA

**Fig. 4:**
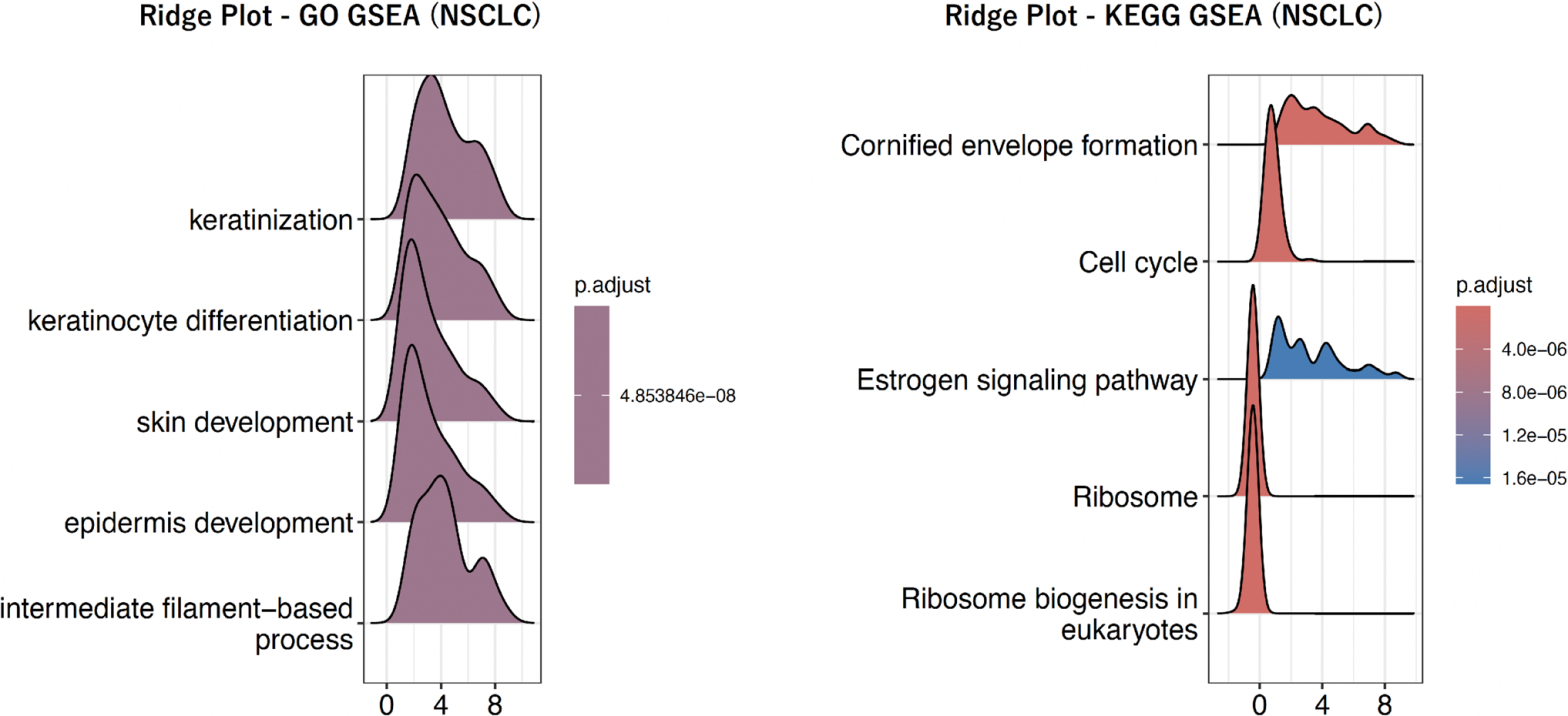
Ridge plot of GSEA for NSCLC classification. Ridge plots of the top gene sets that were most significantly enriched in GO- and KEGG-based GSEA for NSCLC classification. The left panel shows the results of GO-based GSEA, while the right panel shows those of KEGG-based GSEA

**Fig. 5:**
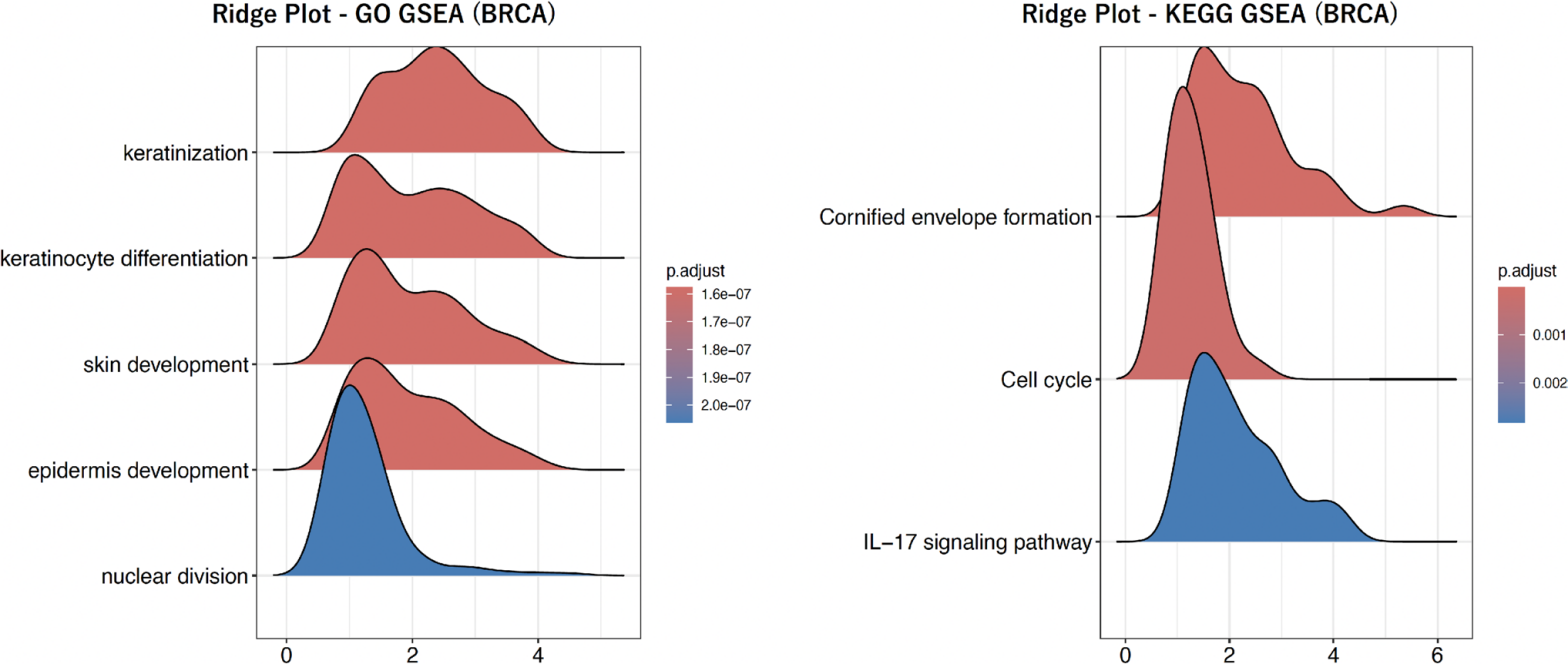
Ridge plot of GSEA for BRCA classification. Ridge plots of the top gene sets that were most significantly enriched in GO- and KEGG-based GSEA for BRCA classification. The left panel shows the results of GO-based GSEA, while the right panel shows those of KEGG-based GSEA

**Fig. 6:**
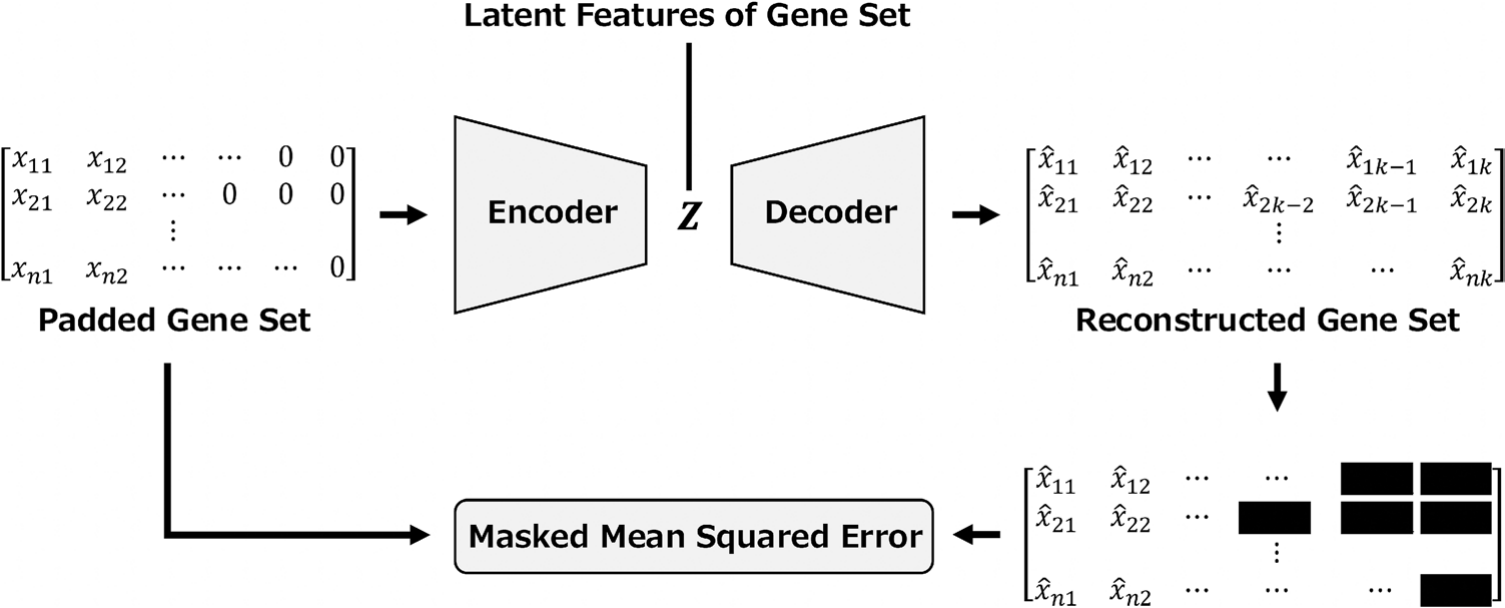
Architecture of the auto encoder used for dimensionality reduction of gene set features. The reconstruction error was calculated using the masked mean squared error because zero-padding was applied to the gene sets to unify the input dimensions

### Dataset

In this study, we used histopathological whole slide images (WSIs) and RNA-seq-derived gene expression profiles from patients with low-grade glioma (LGG), non-small cell lung cancer (NSCLC), and breast cancer (BRCA) obtained from The Cancer Genome Atlas (TCGA). All datasets were downloaded from the Genome Data Commons (https://portal.gdc.cancer.gov/). The LGG dataset consisted of 225 grade II and 242 grade III patients. The NSCLC dataset included 465 lung adenocarcinoma (LUAD) and 476 lung squamous cell carcinoma (LUSC) patients. The BRCA dataset comprised 402 Luminal A and 108 triple-negative breast cancer (TNBC) patients. For each disease dataset, 80% of the samples were used for hyperparameter tuning, and the remaining 20% were used for testing. Hyperparameter tuning was conducted in a stratified five-fold cross-validation.

### Preprocessing

WSIs are a valuable source of information for tumor diagnosis. However, it is impractical to process an entire image at once due to its extremely high resolution. Therefore, WSIs were divided into tiled patches of 256$\times$256 pixels using Histolab [49] in this study. During this process, patches in which the tissue area accounted for more than 90% of the patch were selected as valid patches to exclude background and non-tissue regions. The tissue area of each patch was estimated using Otsu thresholding to separate tissue from background. This criterion effectively removed patches that did not sufficiently contain tumor tissue as shown in Fig. 2. Because the amount of tissue contained in each WSI varies across patients, the number of valid patches also differed among patients. To prevent imbalance in the input data across patients, an equal number of patches was used for each patient in the analysis. For each remaining patch within a WSI, the proportion of tissue area was computed automatically, without manual inspection. In this dataset, the maximum number of valid patches that could be uniformly obtained from all patients was 64. Hence, 64 patches were selected and used for analysis for each patient.

Each patient’s gene expression profile contains a total of 60,660 genes. First, differentially expressed gene (DEG) analysis was performed to evaluate differences in gene expression levels between the two groups. DESeq2 was used for the DEG analysis. Based on these results, Gene Set Enrichment Analysis (GSEA) was applied using Gene Ontology (GO) terms and Kyoto Encyclopedia of Genes and Genomes (KEGG) pathways, respectively. While GO provides comprehensive functional annotations such as molecular functions and biological processes, KEGG offers pathway-level groupings, including metabolic pathways and signaling cascades. Because the biological interpretations of gene sets derived from these resources are inherently different, GO- and KEGG-based gene sets were used independently and were not integrated in this study. GSEA was used to identify gene sets showing statistically significant expression changes between the two groups. In this process, gene sets with an adjusted p-value of less than 0.01 were considered significant. The number of significant gene sets identified for each disease is summarized in Table 1, and representative GSEA results for the most significant gene sets are shown in Fig. 3 for LGG, Fig. 4 for NSCLC, and Fig. 5 for BRCA respectively.

For the gene expression values of genes included in each gene set, TPM normalization and log2 scaling were applied. Before these gene sets were used as inputs to the MIL model, an autoencoder was applied to compress them into low-dimensional latent features so that their dimensionality matched that of the image features. However, it is difficult to directly input multiple gene sets from a single patient into an autoencoder simultaneously because the number of genes contained in each gene set differs. Therefore, zero padding was applied to unify the shapes of the gene sets. Since the padding values are artificially introduced and should not be treated equivalently to true gene expression values, a masked mean squared loss was adopted during autoencoder training. This loss function enables the model to be trained to reconstruct only the actual gene expression values. The latent features obtained from the trained autoencoder were then used as gene set features and served as inputs to the MIL model. Figure 6 shows the architecture and the overall flow of the autoencoder-based approach employed here.

**Table 2:** Comparison of MobileNetV4, ResNet50d, and CTransPath parameter complexity

Model	Total parameters	Parameters size (MB)
Reduced MobileNetV4	68,880	0.26
Original MobileNetV4	340,992	1.3
ResNet50d	23,527,264	89.75
CTransPath	27,496,716	104.89

**Table 3:** Comparison of backbone architectures in an end-to-end MIL model using WSI-only input based on five-fold cross-validation. Classification performance of Original MobileNetV4, MobileNetV4 with reduced UIBs, and MobileNetV4 with both UIB reduction and APoZ pruning (proposed method) is reported

Task	Pruning	Data	Sensitivity	Specificity	F1-score	PR-AUC	ROC-AUC
LGG	Original	WSI	0.815±0.075	0.361±0.129	0.677±0.039	0.698±0.076	0.656±0.088
LGG	UIB	WSI	0.644±0.059	0.595±0.193	0.639±0.043	0.708±0.080	0.663±0.056
LGG	UIB+APoZ	WSI	0.660±0.051	0.600±0.177	0.651±0.051	0.722±0.075	0.685±0.067
NSCLC	Original	WSI	0.995±0.009	0.041±0.039	0.678±0.013	0.693±0.060	0.720±0.041
NSCLC	UIB	WSI	0.787±0.052	0.801±0.025	0.794±0.025	0.873±0.041	0.874±0.018
NSCLC	UIB+APoZ	WSI	0.814±0.070	0.804±0.050	0.811±0.027	0.897±0.011	0.895±0.008
BRCA	Original	WSI	0.081±0.097	0.950±0.049	0.113±0.134	0.381±0.139	0.696±0.050
BRCA	UIB	WSI	0.407±0.047	0.832±0.054	0.401±0.075	0.400±0.146	0.723±0.087
BRCA	UIB+APoZ	WSI	0.511±0.212	0.904±0.022	0.536±0.156	0.514±0.138	0.787±0.061

**Table 4:** Baseline comparison using WSI-only input based on five-fold cross-validation. Classification performance of CLAM, TransMIL, and the proposed end-to-end MIL model with Reduced MobileNetV4 is reported. Proposed MIL in the table denotes Proposed MIL with reduced MobileNetV4

Task	MIL model	Data	Sensitivity	Specificity	F1-score	PR-AUC	ROC-AUC
LGG	CLAM	WSI	0.639±0.070	0.641±0.073	0.638±0.039	0.690±0.077	0.677±0.079
LGG	TransMIL	WSI	0.644±0.146	0.578±0.128	0.631±0.120	0.720±0.075	0.672±0.077
LGG	Proposed MIL	WSI	0.660±0.051	0.600±0.177	0.651±0.051	0.722±0.075	0.685±0.067
NSCLC	CLAM	WSI	0.772±0.056	0.786±0.041	0.777±0.027	0.850±0.045	0.861±0.028
NSCLC	TransMIL	WSI	0.803±0.059	0.766±0.070	0.791±0.024	0.874±0.026	0.872±0.024
NSCLC	Proposed MIL	WSI	0.814±0.070	0.804±0.050	0.811±0.027	0.897±0.011	0.895±0.008
BRCA	CLAM	WSI	0.291±0.053	0.596±0.160	0.385±0.040	0.525±0.077	0.769±0.053
BRCA	TransMIL	WSI	0.256±0.131	0.941±0.026	0.340±0.149	0.461±0.196	0.738±0.099
BRCA	Proposed MIL	WSI	0.511±0.212	0.904±0.022	0.536±0.156	0.514±0.138	0.787±0.061

**Table 5:** Comparison of classification performance across different input modalities based on five-fold cross-validation. The classification performance of the end-to-end MIL model using WSI-only, GO-only, and combined WSI and GO (WSI+GO) inputs is reported

Task	Data	Sensitivity	Specificity	F1-score	PR-AUC	ROC-AUC
LGG	WSI	0.660±0.051	0.600±0.177	0.651±0.051	0.722±0.075	0.685±0.067
LGG	GO	0.562±0.041	0.795±0.087	0.642±0.044	0.778±0.050	0.731±0.064
LGG	WSI+GO	0.722±0.075	0.678±0.119	0.714±0.071	0.798±0.034	0.763±0.053
NSCLC	WSI	0.814±0.070	0.804±0.050	0.811±0.027	0.897±0.011	0.895±0.008
NSCLC	GO	0.932±0.044	0.957±0.066	0.944±0.047	0.960±0.055	0.968±0.032
NSCLC	WSI+GO	0.937±0.061	0.914±0.065	0.927±0.052	0.984±0.019	0.982±0.021
BRCA	WSI	0.511±0.212	0.904±0.022	0.536±0.156	0.514±0.138	0.787±0.061
BRCA	GO	0.848±0.161	0.947±0.047	0.829±0.129	0.802±0.157	0.939±0.052
BRCA	WSI+GO	0.756±0.157	0.966±0.029	0.799±0.060	0.851±0.093	0.955±0.033

**Table 6:** Comparison of classification performance across different input modalities based on five-fold cross-validation. The classification performance of the end-to-end MIL model using WSI-only, KEGG-only, and combined WSI and KEGG (WSI+KEGG) inputs is reported

Task	Data	Sensitivity	Specificity	F1-score	PR-AUC	ROC-AUC
LGG	WSI	0.660±0.051	0.600±0.177	0.651±0.051	0.722±0.075	0.685±0.067
LGG	KEGG	0.624±0.117	0.689±0.096	0.649±0.056	0.770±0.082	0.741±0.049
LGG	WSI+KEGG	0.727±0.062	0.661±0.102	0.712±0.060	0.810±0.028	0.773±0.031
NSCLC	WSI	0.814±0.070	0.804±0.050	0.811±0.027	0.897±0.011	0.895±0.008
NSCLC	KEGG	0.929±0.035	0.951±0.044	0.940±0.032	0.975±0.024	0.970±0.016
NSCLC	WSI+KEGG	0.943±0.048	0.933±0.049	0.939±0.042	0.984±0.020	0.983±0.019
BRCA	WSI	0.511±0.212	0.904±0.022	0.536±0.156	0.514±0.138	0.787±0.061
BRCA	KEGG	0.827±0.109	0.947±0.004	0.818±0.100	0.824±0.180	0.939±0.059
BRCA	WSI+KEGG	0.733±0.148	0.953±0.023	0.766±0.112	0.831±0.114	0.950±0.029

**Fig. 7:**
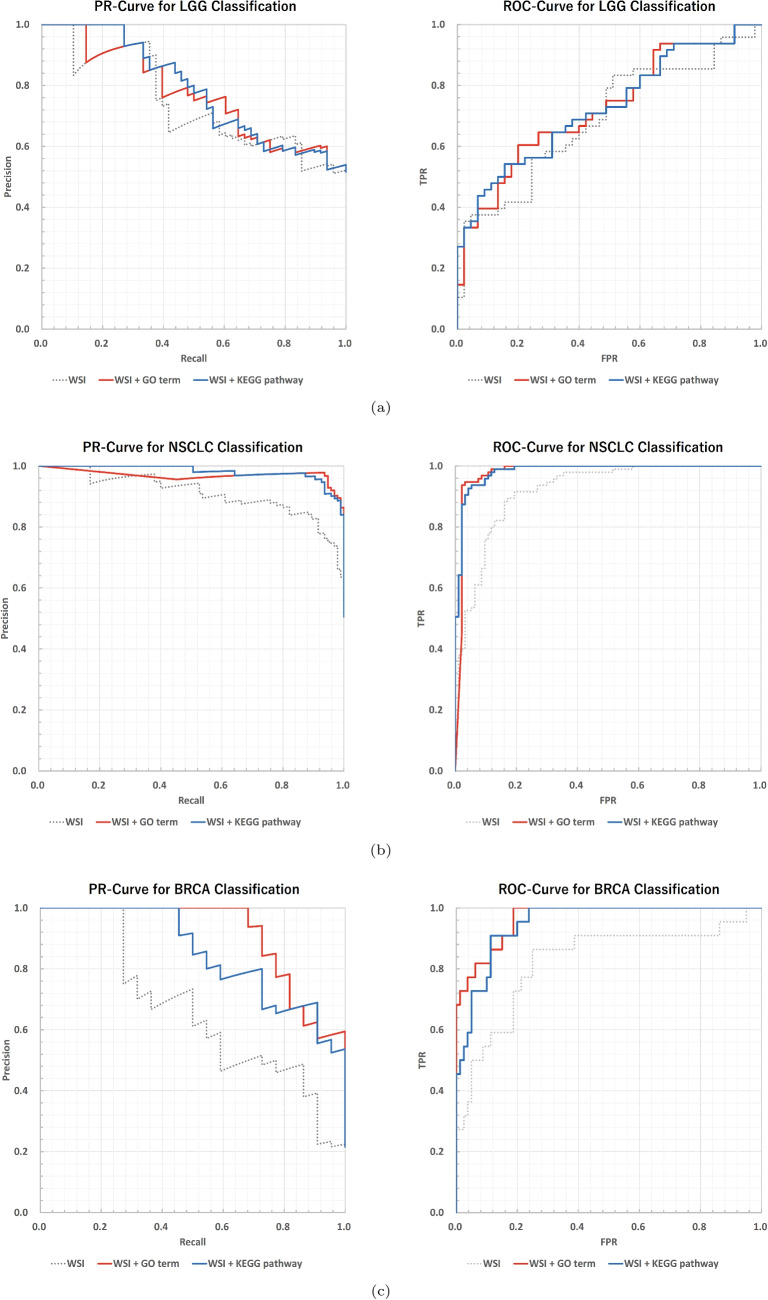
Comparison of PR and ROC curves between modalities on the independent test data for (**a**) LGG, (**b**) NSCLC, and (**c**) BRCA classification. The left panel shows PR-curves, while the right panel shows ROC-curves

**Fig. 8:**
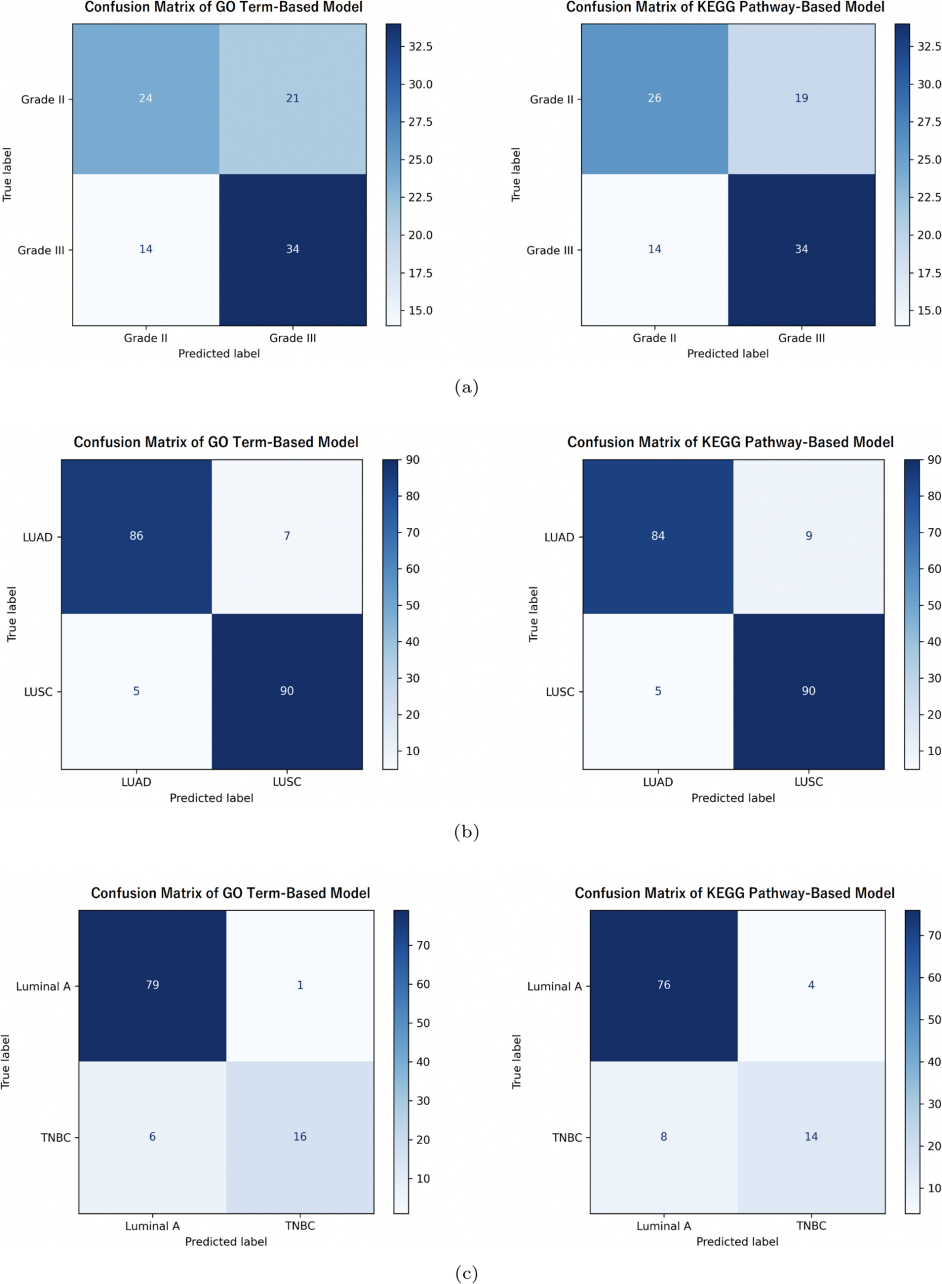
Confusion matrices of proposed model on the independent test data for (**a**) LGG, (**b**) NSCLC, and (**c**) BRCA classification. The left panel shows the results with WSI + GO term, while the right panel shows the results with WSI + KEGG pathway

## Results

In this study, we evaluated the proposed method for three diseases: low-grade glioma (LGG), non-small cell lung cancer (NSCLC), and breast cancer (BRCA). The experiments were conducted on an Ubuntu 20.04 environment equipped with an AMD Ryzen 9 3900X processor, 64 GB of RAM, and an NVIDIA GeForce RTX 4900 GPU. The software environment consisted of Python 3.12, PyTorch 2.5.1, and CUDA 12.1. The number of model parameters and model size of the backbones used in this comparison are summarized in Table 2. The proposed reduced MobileNetV4 is significantly more lightweight than ResNet50d and CTransPath. Despite this, the proposed method achieved higher classification performance than models using ResNet50d or CTransPath as the backbone. In addition, the proposed method reduced GPU memory consumption from 14.9 GiB to 11.6 GiB compared with the MIL model using the Original MobileNetV4, thereby substantially lowering computational resource requirements. On the other hand, no significant difference in execution time was observed between the Original and reduced models. This is likely because the proposed MIL model incorporates multiple attention mechanisms, which constitute the main computational bottleneck. Overall, these results demonstrate that high classification performance can be maintained through end-to-end learning even when a lightweight feature extractor is employed, while substantially reducing the required GPU memory and model size.

### Comparison with Structured Pruning and MIL Models Using WSIs

We compared three configurations of MobileNetV4, which serves as the backbone of the proposed end-to-end MIL model: the Original model, a model with UIB reduction only, and a model with both UIB reduction and APoZ pruning. In the experiments of this section, only WSI data were used as input. The experimental results are summarized in Table 3. Across all tasks, the original model tended to make biased predictions. In particular, it showed markedly low specificity in LGG and NSCLC classification, and markedly low sensitivity in BRCA classification. In contrast, introducing UIB reduction improved the balance between specificity and sensitivity. Furthermore, the proposed method combining UIB reduction and APoZ pruning achieved the highest PR-AUC and ROC-AUC across all tasks. We consider that the performance improvement observed after component removal is largely attributable to the reduction of internal model redundancy. The original MobileNetV4 was designed for large-scale datasets and thus may be overly complex for the binary classification task considered in this study. Note that on the NSCLC task, Original model obtained best sensitivity and worst specificity when compared to the reduced counterparts. This led to the final AUC values of the original model to be below the performance of consistent sensitivity, specificity values obtained by the reduced models. A reverse scenario was observed in the BRCA wherein the Original model obtained worst sensitivity and best specificity when compared to the reduced models. The final AUC values were lower overall for all three tasks indicating that the reduced models obtain promising performance without utilizing large computational cost. The original model did not exhibit overfitting as evidenced by the final F1 scores and AUC values. Overall, the proposed approach integrating UIB reduction and APoZ pruning demonstrated a consistent trend of performance improvement across all cancer classification tasks. In addition, the proposed reduced MobileNetV4 achieved an approximately 80% reduction in the number of model parameters, from 340,992 to 68,880, compared with the Original model.

Next, we compared the proposed end-to-end MIL model with representative MIL methods, namely CLAM and TransMIL. The experimental results are presented in Table 4. In LGG classification, although the proposed method exhibited less stable specificity than CLAM and TransMIL, it achieved slightly higher PR-AUC and ROC-AUC. Paired t-test was performed to check if PR-AUC/ROC-AUC improvements over CLAM and TransMIL are statistically significant. We observed the improvements by our proposed MIL in PR-AUC is statistically insignificant with p-values at 0.335 (against CLAM), and 0.945 (against TransMIL) and also for ROC-AUC with p-values 0.838 (against CLAM), and 0.695 (against TransMIL) respectively. In NSCLC classification, TransMIL outperformed CLAM in terms of PR-AUC and ROC-AUC, while the proposed method surpassed both. In BRCA classification, the proposed method achieved a higher ROC-AUC than CLAM and TransMIL. Although considerable variability in sensitivity was observed, an improvement in F1-score was also noted. Overall, it was confirmed that the proposed model achieved performance comparable to or better than CLAM and TransMIL in terms of PR-AUC and ROC-AUC across the cancer classification tasks. On the other hand, performance variability was observed, particularly in LGG and BRCA classification, suggesting that discrimination based on WSI alone may be challenging. The UIB-reduced model exhibited more stable sensitivity and specificity than the Original model. In addition, the proposed method incorporates both UIB reduction and APoZ pruning, demonstrating higher PR-AUC and ROC-AUC than the other configurations. Although the magnitude of improvement was modest and statistical significance was limited, the proposed MIL with the reduced MobileNetV4 provided improved performance. As noted in the original MobileNetV4 work [13], due to introduction of UIB, highly efficient image feature extraction with better model capacity is possible. Although our approach reduces components including UIBs, we consider that the remaining UIBs still effectively function to preserve efficient feature representations.

### Effect of Multimodal Integration of WSI and Gene Expression

We compared three input configurations: WSI alone, gene sets alone (based on GO terms or KEGG pathways), and the integration of WSI and gene sets. The classification results are shown in Tables 5, and 6 for utilizing GO and KEGG inputs respectively. The corresponding PR and ROC curves are shown in the Fig. 7. The confusion matrix of the integrated model is shown in the Fig. 8. In LGG classification, an improvement in PR-AUC and ROC-AUC was observed compared with WSI alone when using gene sets alone. Furthermore, integrating WSI with gene sets based on GO terms resulted in PR-AUC and ROC-AUC values that exceeded those obtained using either WSI alone or GO-based gene sets alone. Similarly, in the WSI + KEGG configuration, improvements in PR-AUC and ROC-AUC were also confirmed, suggesting complementarity between image information and gene set information. On the other hand, overlapping confidence intervals were observed between the image-only and image-gene set integrated models, indicating that there remains room for improvement. In NSCLC classification, the use of gene sets alone yielded high PR-AUC and ROC-AUC values. In addition, integrating WSI with gene sets further improved PR-AUC and ROC-AUC. In BRCA classification, WSI alone resulted in low PR-AUC and large performance variability. In contrast, PR-AUC and ROC-AUC improved substantially when gene sets were used as input. When WSI and gene sets were integrated, PR-AUC and ROC-AUC were further improved and exhibited more stable values. Across all classification tasks, no substantial performance differences were observed between the integration of WSI with GO-based gene sets and the integration of WSI with KEGG-based gene sets. Overall, these results demonstrate that integrating WSI and gene sets leads to a consistent trend of improvement in PR-AUC and ROC-AUC across all cancer classification tasks.

**Table 7:** Comparison of MIL models with different multimodal aggregation mechanisms using WSI+GO input based on five-fold cross-validation. The classification performance of models using only Self-Attention, Co-Attention models, and the proposed MIL model combining Self-Attention and Cross-Attention is reported. Dual-Attention in the table denotes Self-Attention + Cross-Attention (proposed method)

Task	MIL aggregation	Data	Sensitivity	Specificity	F1-score	PR-AUC	ROC-AUC
LGG	Self-Attention	WSI+GO	0.660±0.063	0.617±0.113	0.654±0.038	0.789±0.043	0.725±0.043
LGG	Co-Attention	WSI+GO	0.541±0.071	0.800±0.051	0.626±0.065	0.788±0.041	0.727±0.039
LGG	Dual-Attention	WSI+GO	0.722±0.075	0.678±0.119	0.714±0.071	0.798±0.034	0.763±0.053
NSCLC	Self-Attention	WSI+GO	0.845±0.063	0.857±0.075	0.852±0.047	0.943±0.039	0.942±0.031
NSCLC	Co-Attention	WSI+GO	0.929±0.027	0.954±0.059	0.942±0.032	0.981±0.021	0.977±0.020
NSCLC	Dual-Attention	WSI+GO	0.937±0.061	0.914±0.065	0.927±0.052	0.984±0.019	0.982±0.021
BRCA	Self-Attention	WSI+GO	0.733±0.064	0.944±0.050	0.757±0.077	0.803±0.131	0.946±0.022
BRCA	Co-Attention	WSI+GO	0.873±0.078	0.941±0.044	0.836±0.075	0.746±0.142	0.944±0.030
BRCA	Dual-Attention	WSI+GO	0.756±0.157	0.966±0.029	0.799±0.060	0.851±0.093	0.955±0.033

**Table 8:** Comparison of MIL models with different multimodal aggregation mechanisms using WSI+KEGG input based on five-fold cross-validation. The classification performance of models using only Self-Attention, Co-Attention models, and the proposed MIL model combining Self-Attention and Cross-Attention is reported. Dual-Attention in the table denotes Self-Attention + Cross-Attention (proposed method)

Task	MIL aggregation	Data	Sensitivity	Specificity	F1-score	PR-AUC	ROC-AUC
LGG	Self-Attention	WSI+KEGG	0.639±0.057	0.639±0.100	0.647±0.042	0.776±0.054	0.713±0.044
LGG	Co-Attention	WSI+KEGG	0.629±0.056	0.728±0.096	0.668±0.019	0.807±0.015	0.747±0.011
LGG	Dual-Attention	WSI+KEGG	0.727±0.062	0.661±0.102	0.712±0.060	0.810±0.028	0.773±0.031
NSCLC	Self-Attention	WSI+KEGG	0.842±0.064	0.847±0.039	0.845±0.027	0.939±0.020	0.938±0.014
NSCLC	Co-Attention	WSI+KEGG	0.932±0.031	0.957±0.040	0.944±0.030	0.981±0.018	0.977±0.016
NSCLC	Dual-Attention	WSI+KEGG	0.943±0.048	0.933±0.049	0.939±0.042	0.984±0.020	0.983±0.019
BRCA	Self-Attention	WSI+KEGG	0.757±0.101	0.941±0.031	0.765±0.075	0.814±0.132	0.944±0.036
BRCA	Co-Attention	WSI+KEGG	0.721±0.193	0.951±0.050	0.751±0.086	0.809±0.111	0.947±0.022
BRCA	Dual-Attention	WSI+KEGG	0.733±0.148	0.953±0.023	0.766±0.112	0.831±0.114	0.950±0.029

**Table 9:** Backbone comparison of the proposed MIL models with WSI+GO input based on five-fold cross-validation. The classification performance of the proposed non-end-to-end MIL model using ResNet50d and CTransPath as feature extractors, and the proposed end-to-end MIL model using Reduced MobileNetV4, is reported

Task	Backbone	Data	Sensitivity	Specificity	F1-score	PR-AUC	ROC-AUC
LGG	ResNet50d	WSI+GO	0.659±0.127	0.605±0.164	0.650±0.104	0.767±0.063	0.719±0.094
LGG	CTransPath	WSI+GO	0.686±0.060	0.605±0.107	0.668±0.036	0.771±0.045	0.721±0.038
LGG	MobileNetV4	WSI+GO	0.722±0.075	0.678±0.119	0.714±0.071	0.798±0.034	0.763±0.053
NSCLC	ResNet50d	WSI+GO	0.906±0.051	0.930±0.072	0.918±0.044	0.976±0.027	0.976±0.023
NSCLC	CTransPath	WSI+GO	0.908±0.063	0.927±0.042	0.918±0.041	0.967±0.048	0.971±0.028
NSCLC	MobileNetV4	WSI+GO	0.937±0.061	0.914±0.065	0.927±0.052	0.984±0.019	0.982±0.021
BRCA	ResNet50d	WSI+GO	0.674±0.113	0.938±0.043	0.707±0.089	0.768±0.140	0.919±0.029
BRCA	CTransPath	WSI+GO	0.710±0.209	0.941±0.035	0.730±0.145	0.794±0.089	0.923±0.035
BRCA	MobileNetV4	WSI+GO	0.756±0.157	0.966±0.029	0.799±0.060	0.851±0.093	0.955±0.033

**Table 10:** Backbone comparison of the proposed MIL models with WSI+KEGG input based on five-fold cross-validation. The classification performance of the proposed non-end-to-end MIL model using ResNet50d and CTransPath as feature extractors, and the proposed end-to-end MIL model using Reduced MobileNetV4, is reported

Task	Backbone	Data	Sensitivity	Specificity	F1-score	PR-AUC	ROC-AUC
LGG	ResNet50d	WSI+KEGG	0.701±0.030	0.661±0.145	0.697±0.049	0.781±0.074	0.737±0.071
LGG	CTransPath	WSI+KEGG	0.671±0.031	0.644±0.095	0.671±0.030	0.793±0.035	0.742±0.039
LGG	MobileNetV4	WSI+KEGG	0.727±0.062	0.661±0.102	0.712±0.060	0.810±0.028	0.773±0.031
NSCLC	ResNet50d	WSI+KEGG	0.927±0.049	0.925±0.070	0.927±0.036	0.967±0.060	0.975±0.035
NSCLC	CTransPath	WSI+KEGG	0.911±0.044	0.917±0.019	0.914±0.025	0.970±0.035	0.968±0.032
NSCLC	MobileNetV4	WSI+KEGG	0.943±0.048	0.933±0.049	0.939±0.042	0.984±0.020	0.983±0.019
BRCA	ResNet50d	WSI+KEGG	0.362±0.126	0.978±0.017	0.497±0.144	0.745±0.170	0.919±0.053
BRCA	CTransPath	WSI+KEGG	0.606±0.131	0.954±0.023	0.678±0.096	0.759±0.092	0.915±0.019
BRCA	MobileNetV4	WSI+KEGG	0.733±0.148	0.953±0.023	0.766±0.112	0.831±0.114	0.950±0.029

### Comparison with Multimodal MIL Design

To evaluate the proposed aggregation mechanism, we compared three approaches: Self-Attention alone, Co-Attention based on MCAT [50], which is a state-of-the-art multimodal aggregation method, and Dual-Attention that combines Self-Attention and Cross-Attention. The classification results are presented in Tables 7 and 8 for utilizing GO and KEGG inputs respectively. In LGG classification, the proposed method achieved higher PR-AUC and ROC-AUC than both Self-Attention alone and Co-Attention, and sensitivity was also substantially improved. Paired t-test was performed to check if PR-AUC/ROC-AUC improvements over Self-Attention and Co-Attention are statistically significant. We observed the improvements by our proposed Dual-Attention in PR-AUC is statistically insignificant with p-values at 0.714 (against Self-Attention), and 0.619 (against Co-Attention) and also for ROC-AUC with p-values 0.296 (against Self-Attention), and 0.246 (against Co-Attention) respectively. In NSCLC classification, the proposed method showed only marginal improvements compared to Self-Attention alone or Co-Attention, with statistical significance being limited. In BRCA classification, ROC-AUC showed comparable results across methods. However, the proposed method achieved a substantially higher PR-AUC. In addition, performance variability remained a challenge for all methods. Overall, these results confirm that the proposed approach, which applies Self-Attention and Cross-Attention to each modality, demonstrates stable aggregation performance.

To evaluate the proposed end-to-end learning framework, we compared the proposed method with non-end-to-end approaches in which the feature extractor is decoupled from the classifier. For the non-end-to-end approaches, we employed feature extractors based on ResNet50d, which has been widely used in previous studies, and CTransPath, which is known as a foundation model. The classification results are shown in Tables 9 and 10 for utilizing GO and KEGG inputs respectively. In LGG classification, the proposed method achieved a slight improvement in PR-AUC compared with ResNet50d and CTransPath, while a substantial improvement was observed in ROC-AUC. In NSCLC classification, both PR-AUC and ROC-AUC were slightly improved. In BRCA classification, PR-AUC and ROC-AUC were substantially improved. Therefore, the proposed model, which adopts a reduced MobileNetV4 as the backbone and is optimized in an end-to-end manner, demonstrated higher classification performance than the non-end-to-end MIL models. This suggests that end-to-end learning may contribute to improved discriminative performance.

**Table 11:** Comparison of classification performance with different gene representation schemes based on five-fold cross-validation. The classification performance of the end-to-end MIL model using WSI and individual gene vectors (WSI+individual gene), WSI and GO (WSI+GO), and WSI and KEGG (WSI+KEGG) as inputs is reported

Task	Data	Sensitivity	Specificity	F1-score	PR-AUC	ROC-AUC
LGG	WSI+Individual gene	0.644±0.051	0.678±0.151	0.665±0.069	0.772±0.095	0.728±0.092
LGG	WSI+GO	0.722±0.075	0.678±0.119	0.714±0.071	0.798±0.034	0.763±0.053
LGG	WSI+KEGG	0.727±0.062	0.661±0.102	0.712±0.060	0.810±0.028	0.773±0.031
NSCLC	WSI+Individual gene	0.903±0.043	0.863±0.109	0.888±0.064	0.962±0.040	0.959±0.042
NSCLC	WSI+GO	0.937±0.061	0.914±0.065	0.927±0.052	0.984±0.019	0.982±0.021
NSCLC	WSI+KEGG	0.943±0.048	0.933±0.049	0.939±0.042	0.984±0.020	0.983±0.019
BRCA	WSI+Individual gene	0.650±0.159	0.963±0.035	0.723±0.126	0.792±0.193	0.928±0.066
BRCA	WSI+GO	0.756±0.157	0.966±0.029	0.799±0.060	0.851±0.093	0.955±0.033
BRCA	WSI+KEGG	0.733±0.148	0.953±0.023	0.766±0.112	0.831±0.114	0.950±0.029

To evaluate the proposed gene feature representations, we compared a single-gene expression vector, GO-based gene set representations, and KEGG-based gene set representations. The classification results are shown in Table 11. In LGG and BRCA classification, the gene set representations achieved higher values for all metrics except specificity. In NSCLC classification, the gene set representations outperformed the single-gene expression vector across all evaluation metrics. Overall, these results indicate a consistent trend toward improved classification performance when using gene representations aggregated based on GO terms or KEGG pathways, compared with single-gene vector representations.

### Performance on Independent Test Set

Tables 1 to 9 provided in Supplementary summarizes the results of all comparative experiments evaluated on the independent test set. The outcomes on the independent test set were generally consistent with the trends observed in cross-validation. In experiments using WSI alone, the application of UIB reduction and APoZ pruning led to improved classification performance. In addition, a tendency toward slightly higher performance compared with existing methods was observed. In experiments integrating both WSI and gene expression profile, the proposed end-to-end multimodal MIL model demonstrated consistent performance improvements over single-modality models, alternative aggregation strategies, and other gene representation approaches.

## Discussion

### Assessment of Classification Performance

#### Effect of Backbone Optimization and Comparison with WSI-based MIL Models

In this study, we adopted MobileNetV4 as the backbone of an end-to-end MIL model and evaluated the effectiveness of UIB reduction and APoZ pruning across multiple cancer classification tasks. The Original model exhibited biased predictions in all tasks. However, introducing UIB reduction improved the balance of predictions. Furthermore, the combination of UIB reduction and APoZ pruning achieved the highest PR-AUC and ROC-AUC across all tasks. These results suggest that the reduced UIBs and the channels in the final convolutional layer may be redundant for the type of few-class classification addressed in this study. In contrast, the performance gains in LGG classification were limited, which may be attributable to the small differences between malignancy grades. In addition, the proposed reduced MobileNetV4 substantially decreased the number of model parameters while maintaining or improving performance, demonstrating its utility from a computational efficiency perspective. Taken together, these findings indicate that structural lightweighting through the combination of UIB reduction and APoZ pruning represents an effective design choice for WSI-based cancer classification.

In comparison with representative MIL methods, namely CLAM and TransMIL, the proposed model achieved PR-AUC and ROC-AUC that were comparable to or higher than those of the existing approaches. Notably, in the NSCLC classification task, the proposed model consistently outperformed both CLAM and TransMIL, suggesting that end-to-end optimization functioned effectively. In contrast, improvements in PR-AUC and ROC-AUC for LGG classification were limited, and variability in specificity was observed. This outcome implies that the biological differences between LGG malignancy grades may be difficult to capture using WSI alone. In the BRCA classification task, while the proposed model substantially outperformed the other models in terms of F1-score and ROC-AUC, it exhibited greater variability in sensitivity. This variability may stem from differences in the number of cases and the diversity of tumor morphologies. Overall, these results demonstrate that the proposed model provides competitive performance relative to existing MIL methods; however, they also highlight persistent limitations of classification based solely on WSI, suggesting the need for extensions incorporating additional sources of information. Furthermore, to avoid imbalance in the amount of image information across patients, this study employed a design in which an equal number of patches was sampled from each WSI. Investigation of the effects of patch number and sampling strategies on model performance lies beyond the primary scope of this study and is left for future work.

#### Effect of Multimodal Integration

In the proposed model, we evaluated the effectiveness of multimodal integration by comparing performance across three settings: WSI alone, gene sets alone, and the integration of WSI and gene sets. The results demonstrated a consistent tendency for PR-AUC and ROC-AUC to improve across all cancer classification tasks when WSI and gene sets were integrated. In the LGG classification task, gene sets alone achieved higher performance than WSI alone, and further performance gains were observed when WSI and gene sets were combined. This finding suggests that image information and gene set information may play complementary roles. However, overlapping confidence intervals were observed between the WSI-only and integrated models, indicating that further validation is required to assess the stability of the performance improvements. In the NSCLC classification task, gene sets alone yielded high classification performance, indicating a substantial contribution of genomic information. Moreover, additional performance improvements were achieved by integrating WSI, suggesting that image information provides complementary benefits. In the BRCA classification task, while WSI alone exhibited large performance variability, the incorporation of gene sets improved stability and led to a notable increase in PR-AUC. TNBC is known to exhibit high heterogeneity compared with Luminal A in terms of both tumor morphology and molecular characteristics, which may make it difficult to consistently extract discriminative features when relying on a single modality. To address this, the proposed model emphasizes features that contribute to classification from both histopathological images and gene expression through self-attention and cross-attention mechanisms. As a result, TNBC-specific information is complementarily integrated across modalities, leading to improved detection performance for the minority TNBC class.

Furthermore, no substantial performance differences were observed between gene sets based on GO terms and those based on KEGG pathways across all classification tasks, indicating that both types of gene representations function effectively. Overall, although genomic information exerts a strong influence, the multimodal approach that integrates WSI and gene sets provides consistent and stable performance improvements across cancer classification tasks, demonstrating its effectiveness. While this study focused on WSI and gene expression profiles, a variety of additional modalities, such as DNA methylation profiles and MRI, have become increasingly available in recent years. Broader multimodal analyses integrating these diverse data sources are expected to more accurately capture the spatial and molecular heterogeneity of tumors, and validation using other types of data remains an important direction for future work.

#### Effect of MIL Aggregation and End-to-End Design

To investigate the impact of different aggregation mechanisms in multimodal MIL on classification performance, we compared models using self-attention only, co-attention, and a dual-attention scheme that combines self-attention and cross-attention. The results showed that the proposed dual-attention consistently exhibited improvements in PR-AUC and ROC-AUC across all cancer classification tasks. The observed performance gains of dual-attention are attributed to the introduction of cross-attention, which enables mutual referencing between image features and gene features. When using self-attention alone, the model is limited to emphasizing important features within each modality and may fail to sufficiently capture inter-modality relationships. In contrast, aggregation via cross-attention facilitates mutual referencing between modalities that contribute to classification, leading to improved performance. Comparison with co-attention is also important for clarifying the positioning of the proposed method. Co-attention has been recognized as an effective approach for capturing inter-modality relationships and demonstrated high classification performance in our experiments as well. However, the proposed dual-attention introduces bidirectional cross-attention between image information and gene information. This design enables more effective aggregation for classification, resulting in performance comparable to or exceeding that of co-attention. Overall, these findings indicate that the proposed approach combining self-attention and cross-attention can serve as an effective aggregation mechanism for multimodal MIL. Nevertheless, performance variability remains an issue, and further investigation is required to improve stability.

We further evaluated the effectiveness of end-to-end optimization by comparing the proposed end-to-end MIL model with non-end-to-end MIL models. The results showed that the proposed approach, which employs the reduced MobileNetV4 as its backbone, exhibited consistent performance improvements across all cancer classification tasks compared with non-end-to-end models using ResNet50d or CTransPath as feature extractors. In particular, substantial improvements in PR-AUC and ROC-AUC were observed for the BRCA classification task, suggesting that jointly optimizing feature extraction, aggregation, and classification in an end-to-end manner may be especially effective for tasks characterized by high diversity in tumor morphology. Moreover, despite employing a significantly lighter backbone than ResNet50d or CTransPath, the proposed method achieved comparable or superior classification performance. These results suggest that task-adaptive feature representations can be learned through end-to-end training without relying on large, pre-trained feature extractors. Overall, these findings demonstrate that end-to-end learning, even with lightweight feature extractors, can achieve a favorable balance between high classification performance and reduced computational resources.

To validate the effectiveness of the proposed gene set representations, we compared single-gene vector representations with gene set representations based on GO terms and KEGG pathways. The results showed that gene set representations achieved higher classification performance than single-gene representations for all metrics except specificity in LGG and BRCA classification, and for all evaluation metrics in NSCLC classification. These findings suggest that representing gene information based on biological functions and pathways, rather than using single-gene-level information directly, may yield feature representations that are more suitable for classification. Furthermore, no substantial differences were observed between gene set representations based on GO terms and those based on KEGG pathways, with both exhibiting stable classification performance. This result indicates that the effectiveness of gene set representations in multimodal MIL does not strongly depend on a specific knowledge base, but rather on the gene set abstraction itself. Taken together, these results suggest that integrating image features with biologically informed gene features and optimizing them in an end-to-end manner is an effective strategy for achieving stable classification performance in multimodal MIL for cancer classification.

### Gene Set-Level Analysis of Attention Weights

We analyzed which gene sets (GO terms or KEGG pathways) were emphasized by the proposed model through its self-attention and cross-attention mechanisms. As an analysis procedure, attention weights assigned to each gene set were first extracted for patients in the test dataset. These values were then aggregated on a per-gene set basis to construct a dataframe of the form [Patient ID] $\times$ [Attention weight, Label]. A two-group comparison using the Mann-Whitney U test was applied to this aggregated data to identify gene sets that were preferentially weighted by the attention mechanism. Statistical significance was determined using FDR-corrected p-values with a threshold of p < 0.01.

Furthermore, to confirm that these group-wise differences were not attributable to random variation, an additional permutation test was conducted for gene sets identified as significant by the Mann-Whitney U test. Specifically, patient labels were randomly shuffled while keeping the trained model and input features fixed, and the group-wise differences in attention weights were recalculated. As a result, the observed group-wise differences were scarcely reproduced under label permutation. These findings suggest that the attention weights reflect non-random patterns based on the correspondence between disease labels and input features.

#### GO Term-Based Analysis

In the Self-Attention analysis of LGG classification, terms such as *protein-DNA complex assembly* (GO:0065004), *regulation of attachment of mitotic spindle microtubules to kinetochore* (GO:1902423), and *regulation of chromosome segregation* (GO:0051983) were significantly enriched. This suggests aberrant activation of mitotic and chromosomal segregation processes, which may reflect dysregulated cell cycle activity. In addition, *positive regulation of leukocyte cell-cell adhesion* (GO:1903039) indicated enhanced immune cell interactions, while *endothelial cell apoptotic process* (GO:0072577) suggested activation of vascular remodeling. In the Cross-Attention analysis, *meiotic chromosome segregation* (GO:0045132) and *DNA-templated DNA replication maintenance of fidelity* (GO:0045005) were significant. This indicates associations with abnormal cell cycle regulation and genomic instability. Meanwhile, *embryonic cranial skeleton morphogenesis* (GO:0048701) and *negative regulation of leukocyte apoptotic process* (GO:2000107) implied reactivation of developmental signaling and promotion of immune cell survival.

In NSCLC classification, the Self-Attention analysis identified significant MHC-related terms (GO:0002396, GO:0002399, GO:0002501, GO:0002503), reflecting differences in antigen presentation pathways between LUAD and LUSC. The enrichment of *nuclear division* (GO:0000280) was consistent with the high proliferative activity and genomic instability characteristic of LUSC. In the Cross-Attention analysis, peptidase activity regulation terms (GO:0052548, GO:0052547, GO:0010466) were significant. This suggests correspondence between protease dynamics in the tumor microenvironment and tissue architecture. Additionally, *skin development* (GO:0043588) and *regulation of mitotic sister chromatid segregation* (GO:0033047) were associated with squamous differentiation and cell cycle progression.

In BRCA classification, Self-Attention analysis revealed significant enrichment of *chromosome segregation* (GO:0007059), *rRNA processing* (GO:0006364), and *ribosome biogenesis* (GO:0042254), reflecting chromosomal instability and elevated metabolic activity characteristic of TNBC. In the Cross-Attention analysis, *organelle fission* (GO:0048285) and *meiotic cell cycle* (GO:0051321) were significantly enriched. This suggests a correspondence between proliferative potential and morphological atypia observed in WSIs. Furthermore, the significance of *skin development* (GO:0043588) and *protein secretion* (GO:0009306) indicated reactivation of ectopic differentiation programs and possible associations with immune cell infiltration and angiogenesis.

**Fig. 9:**
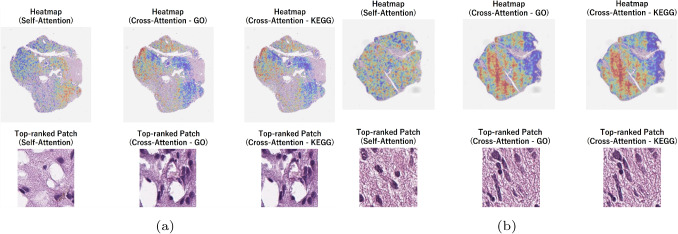
Visualization of Self-Attention and Cross-Attention mechanisms for (**a**) LGG II, and (**b**) LGG III histopathological images. The upper heatmaps show the spatial distribution of attention weights on the WSI obtained from the self-attention and cross-attention mechanisms (gene features based on GO/KEGG were used as a query)

**Fig. 10:**
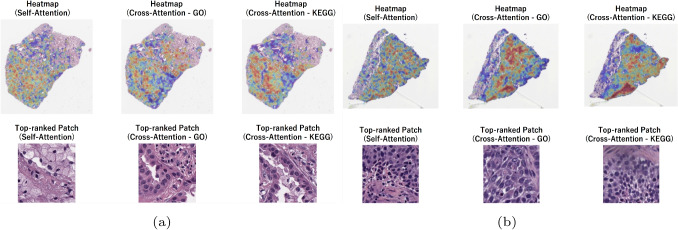
Visualization of Self-Attention and Cross-Attention mechanisms for (**a**) LUAD, and (**b**) LUSC) histopathological images. The upper heatmaps show the spatial distribution of attention weights on the WSI obtained from the self-attention and cross-attention mechanisms (gene features based on GO/KEGG were used as a query)

**Fig. 11:**
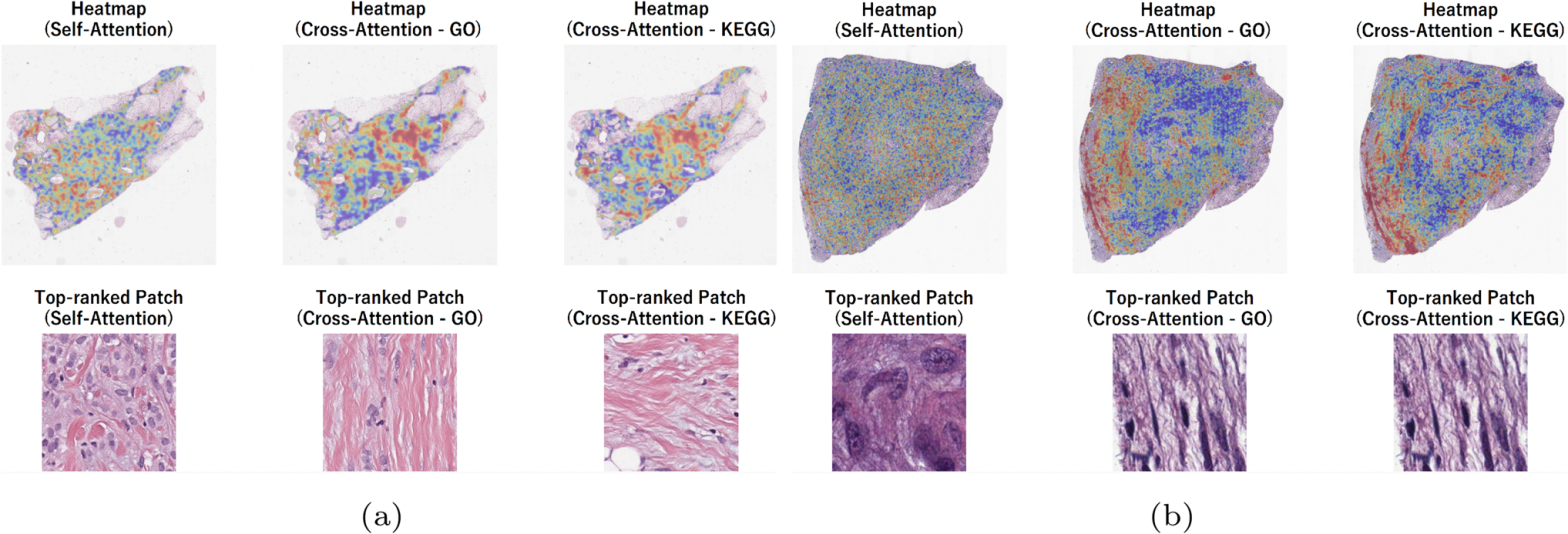
Visualization of Self-Attention and Cross-Attention mechanisms for (**a**) Luminal A, and (**b**) TNBC histopathological images. The upper heatmaps show the spatial distribution of attention weights on the WSI obtained from the self-attention and cross-attention mechanisms (gene features based on GO/KEGG were used as a query)

#### KEGG Pathway-Based Analysis

In the LGG classification, the Self-Attention analysis revealed significant enrichment in *Cell cycle* (hsa04110), *Antigen processing and presentation* (hsa04612), *Phosphatidylinositol signaling system* (hsa04070), *Proteoglycans in cancer* (hsa05205), and *Transcriptional misregulation in cancer* (hsa05202). The enrichment of hsa04110 suggests dysregulated cell cycle control and enhanced proliferative activity in Grade III tumors, while hsa04070 indicates increased invasiveness and therapeutic resistance. Furthermore, hsa05205 and hsa05202 are involved in tumor cell plasticity and invasion promotion, and hsa04612 likely reflects the establishment of an immunosuppressive microenvironment through immune evasion mechanisms. In the Cross-Attention analysis, *Rheumatoid arthritis* (hsa05323), *Transcriptional misregulation in cancer* (hsa05202), *Allograft rejection* (hsa05330), *Circadian entrainment* (hsa04713), and *Amphetamine addiction* (hsa05031) were significantly enriched. These pathways suggest the presence of chronic inflammation, immune dysregulation, and circadian rhythm disruption - metabolic and immunological abnormalities characteristic of progressive LGG.

In the NSCLC classification, the Self-Attention analysis identified significant enrichment in *Complement and coagulation cascades* (hsa04610), *Cornified envelope formation* (hsa04382), *Staphylococcus aureus infection* (hsa05150), *Asthma* (hsa05310), and *Allograft rejection* (hsa05330). The enrichment of hsa04610 reflects the inflammatory tumor microenvironment and increased immune infiltration characteristic of LUSC, while hsa04382 represents discriminative learning of epithelial differentiation-related gene expression. The significance of immune-related pathways indicates that the model captured transcriptional differences in immune activation states between LUAD and LUSC. In the Cross-Attention analysis, *Ribosome biogenesis in eukaryotes* (hsa03008), *Asthma* (hsa05310), *Allograft rejection* (hsa05330), *Nicotine addiction* (hsa05033), and *Complement and coagulation cascades* (hsa04610) were significantly enriched. This suggests that the model learned correspondences between molecular signatures such as proliferative capacity, immune response, and environmental factors (e.g., smoking) and tissue structure.

In the BRCA cancer classification, *Cornified envelope formation* (hsa04382) was significantly enriched in the Self-Attention analysis, reflecting the elevated expression of keratin-related genes characteristic of TNBC. In the Cross-Attention analysis, both hsa04382 and *IL-17 signaling pathway* (hsa04657) were significant. This suggests that the model linked keratinization tendencies and altered cell-cell adhesion with activation of the inflammatory tumor microenvironment.

### Role of Attention Mechanisms in Multimodal Integration

The experimental results indicate that the proposed attention mechanisms assign relatively higher weights to gene sets and histopathological image regions that contribute to cancer classification. In this study, attention weights are not regarded as direct biological indicators but are interpreted as reflecting elements emphasized by the model during the classification process. In the self-attention-based analysis, gene sets related to cell cycle regulation and immune responses were assigned relatively higher attention weights. In contrast, the cross-attention-based analysis tended to selectively emphasize gene sets that exhibited stronger associations with image-derived features. These findings suggest that the attention mechanisms capture feature patterns that are informative for classification both within individual modalities and across modalities.

In Fig. 9 we show an example WSI from each LGG II and LGG III classes with the spatial distribution of attention weights as well as showcasing the top-ranked patch with respect to Self-Attention, Cross-Attention - GO, and Cross-Attention - KEGG. A similar visualization is provided for LUAD and LUSC in Fig. 10, and in Fig. 11 for Luminal A and TNBC classes. The top row show heatmaps that visualize the attention weights assigned to patch-level image features on WSI by the self-attention and cross-attention mechanisms of the proposed model. These correspond to the self-attention weights, the cross-attention weights when gene features based on GO terms were used as a query, and the cross-attention weights when gene features based on KEGG pathways were used as a query. The lower row show the patch images with the highest attention weights. These visualizations serve as qualitative examples highlighting differences in the regions emphasized by distinct attention mechanisms and are not intended to provide definitive identification of specific histopathological features. Across disease tasks, differences were observed in the regions emphasized by self-attention and cross-attention, and different tendencies were observed when comparing high-attention image patches. These differences likely reflect the distinct roles of the two mechanisms. Self-attention highlights regions that are informative for classification based solely on image features. In contrast, cross-attention references image features using gene-derived representations as queries and relatively emphasizes morphological patterns that may correspond to molecular-level information. Together, these observations suggest that the combined use of self-attention and cross-attention enhances classification performance. The primary objective of this study is to enhance classification performance through effective multimodal integration rather than to derive direct biological interpretations from attention weights. While attention heatmaps may serve as auxiliary tools for hypothesis generation, establishing their biological validity requires experimental validation, including expert pathological assessment.

### Limitations

This study has a few limitations. First, the datasets were exclusively derived from TCGA. Although cross-validation and evaluation using independent test data were performed, demonstrating clinical utility requires verification of generalization performance using datasets from multiple institutions. In addition, inconsistencies such as variations in staining conditions of histopathological slides and differences in gene expression profiling procedures should also be taken into consideration. To address these issues, the application of standardization techniques, such as stain normalization across institutions, would be effective. Second, calibration analysis to assess the reliability of predicted probabilities was not performed in this study. In our proposed approach, we utilized the reduced MobileNetV4, and as noted in the original work [13] due to introduction of UIB, highly efficient image feature extraction with better model capacity is possible which can help alleviate the issues such as batch effects and possible stain variations across sites. For clinical application, evaluating the validity of probabilistic predictions is important, and this aspect will be investigated in future work. Furthermore, this study did not include comparisons with advanced genomics models on the molecular side, such as graph-based or point-cloud-based approaches. The primary objective of this study is not to design detailed representations of individual gene structures, but rather to evaluate the effectiveness of a multimodal MIL framework that integrates histopathological images and gene expression data. Accordingly, we adopted biologically curated gene sets as the basic unit of representation, and direct comparisons or integration with more advanced molecular models remain subjects for future investigation. Finally, collaborative evaluation between the attention mechanisms and clinical experts is essential to make the proposed model more clinically interpretable. Moving beyond attention heatmaps to a quantitative measurements (e.g. mean attention score) require ground truth region of interest (ROI) annotations which are unfortunately not available for TCGA datasets TCGA-LGG, TCGA-NSCLC, and TCGA-BRCA considered here [51]. Though the attention mechanism provides meaningful insights intrinsically [52], obtaining post-hoc explanations remains challenging. To achieve this, we must implement visualization systems for inference processes and weighting, and engage in evaluation by clinical specialists. In the future, through these extensions, multimodal analysis is expected to contribute not only to improved classification performance but also to a deeper understanding of tumors and the discovery of novel therapeutic targets. In particular, the pathway-level importance analysis derived from attention weights represents a promising research approach that bridges data-driven methodologies with pathological knowledge.

## Conclusion

In this study, we proposed a lightweight end-to-end multimodal MIL model that integrates histopathological images and gene expression profiles. The proposed method enables comprehensive analysis of both morphological and molecular information by combining efficient image feature extraction using a reduced MobileNetV4 with gene set-level representations based on Gene Set Enrichment Analysis (GSEA). Experimental results showed that our method outperformed image-only models and conventional single-vector gene input models in classifying low-grade glioma, non-small cell lung cancer, and breast cancer. In particular, the notable improvement in PR-AUC for breast cancer indicates enhanced detection capability in imbalanced datasets. These findings confirm that integrating image and gene expression data is effective for accurately capturing morphological and molecular differences across diseases. Furthermore, our lightweight architecture employed maintains high classification performance while reducing computational resources, making it well-suited for large-scale pathological data analysis and potential clinical applications. Future work includes extending the model to a broader range of diseases, incorporating additional modalities, and enhancing the interpretability of the attention mechanism.

## Supplementary Information

Below is the link to the electronic supplementary material.

Supplementary file 1 (pdf 45 KB)

## Data Availability

All datasets utilized in the paper are available publicly from the TCGA Genome Data Commons (https://portal.gdc.cancer.gov/).
