# Supplementary material for: End-to-End Multimodal Multiple Instance Learning for Cancer Histopathology Classification with Dual-Attention Fusion
Source: J Med Syst. 2026 Apr 15;50(1):54. doi: 10.1007/s10916-026-02379-0 (PMC13083524; doi:10.1007/s10916-026-02379-0)
Supplement: Supplementary file 1 — (pdf 45 KB) [file 10916_2026_2379_MOESM1_ESM.pdf]

Supplementary file for "End-to-End Multimodal Multiple Instance Learning for Cancer Histopathology Classification with Dual-Attention Fusion"

The following Tables 1 to 9 show comparative experiments evaluated on the independent test set.

Table 1: Comparison of backbone architectures in an end-to-end MIL model using WSI-only input based on independent testing. Classification performance of Original MobileNetV4, MobileNetV4 with reduced UIBs, and MobileNetV4 with both UIB reduction and APoZ pruning (proposed method) is reported.

Task	Pruning	Data	Sensitivity	Specificity	F1-score	PR-AUC	ROC-AUC
LGG	Original	WSI	0.846	0.333	0.688	0.683	0.638
LGG	UIB	WSI	0.590	0.750	0.648	0.717	0.677
LGG	UIB+APoZ	WSI	0.641	0.722	0.676	0.749	0.697
NSCLC	Original	WSI	0.987	0.014	0.673	0.718	0.727
NSCLC	UIB	WSI	0.816	0.797	0.810	0.876	0.871
NSCLC	UIB+APoZ	WSI	0.816	0.787	0.805	0.905	0.886
BRCA	Original	WSI	0.111	0.938	0.167	0.399	0.709
BRCA	UIB	WSI	0.471	0.781	0.410	0.400	0.779
BRCA	UIB+APoZ	WSI	0.545	0.888	0.558	0.623	0.800

Table 2: Baseline comparison using WSI-only input based on independent testing. Classification performance of CLAM, TransMIL, and the proposed end-to-end MIL model with Reduced MobileNetV4 is reported. Proposed MIL in the table denotes Proposed MIL with Reduced MobileNetV4.

Task	MIL model	Data	Sensitivity	Specificity	F1-score	PR-AUC	ROC-AUC
LGG	CLAM	WSI	0.615	0.632	0.623	0.707	0.691
LGG	TransMIL	WSI	0.615	0.639	0.632	0.714	0.697
LGG	Proposed MIL	WSI	0.641	0.722	0.676	0.749	0.697
NSCLC	CLAM	WSI	0.737	0.836	0.783	0.857	0.871
NSCLC	TransMIL	WSI	0.868	0.689	0.800	0.869	0.879
NSCLC	Proposed MIL	WSI	0.816	0.787	0.805	0.905	0.886
BRCA	CLAM	WSI	0.318	0.636	0.424	0.592	0.793
BRCA	TransMIL	WSI	0.364	0.888	0.410	0.431	0.776
BRCA	Proposed MIL	WSI	0.545	0.888	0.558	0.623	0.800

Table 3: Comparison of classification performance across different input modalities based on independent testing. The classification performance of the end-to-end MIL model using WSI-only, GO-only, and combined WSI and GO (WSI+GO) inputs is reported.

Task	Data	Sensitivity	Specificity	F1-score	PR-AUC	ROC-AUC
LGG	WSI	0.641	0.722	0.676	0.749	0.697
LGG	GO	0.658	0.694	0.676	0.774	0.723
LGG	WSI+GO	0.688	0.578	0.660	0.779	0.740
NSCLC	WSI	0.816	0.787	0.805	0.905	0.886
NSCLC	GO	0.916	0.968	0.941	0.929	0.954
NSCLC	WSI+GO	0.947	0.935	0.942	0.969	0.978
BRCA	WSI	0.545	0.888	0.558	0.623	0.800
BRCA	GO	0.682	0.962	0.750	0.853	0.938
BRCA	WSI+GO	0.682	0.962	0.750	0.901	0.966

Table 4: Comparison of classification performance across different input modalities based on independent testing. The classification performance of the end-to-end MIL model using WSI-only, KEGG-only, and combined WSI and KEGG (WSI+KEGG) inputs is reported.

Task	Data	Sensitivity	Specificity	F1-score	PR-AUC	ROC-AUC
LGG	WSI	0.641	0.722	0.676	0.749	0.697
LGG	KEGG	0.513	0.778	0.597	0.708	0.711
LGG	WSI+KEGG	0.688	0.533	0.647	0.783	0.725
NSCLC	WSI	0.816	0.787	0.805	0.905	0.886
NSCLC	KEGG	0.874	0.946	0.907	0.965	0.964
NSCLC	WSI+KEGG	0.947	0.903	0.928	0.981	0.982
BRCA	WSI	0.545	0.888	0.558	0.623	0.800
BRCA	KEGG	0.727	0.975	0.800	0.857	0.937
BRCA	WSI+KEGG	0.682	0.938	0.714	0.843	0.951

Table 5: Comparison of MIL models with different multimodal aggregation mechanisms using WSI+GO input based on independent testing. The classification performance of models using only Self-Attention, Co-Attention models, and the proposed MIL model combining Self-Attention and Cross-Attention is reported. Dual-Attention in the table denotes Self-Attention + Cross-Attention (proposed method).

Task	MIL Aggregation	Data	Sensitivity	Specificity	F1-score	PR-AUC	ROC-AUC
LGG	Self-Attention	WSI+GO	0.737	0.444	0.651	0.774	0.715
LGG	Co-Attention	WSI+GO	0.590	0.861	0.687	0.779	0.716
LGG	Dual-Attention	WSI+GO	0.688	0.578	0.660	0.779	0.740
NSCLC	Self-Attention	WSI+GO	0.922	0.973	0.947	0.969	0.971
NSCLC	Co-Attention	WSI+GO	0.908	0.919	0.914	0.967	0.964
NSCLC	Dual-Attention	WSI+GO	0.947	0.935	0.942	0.969	0.978
BRCA	Self-Attention	WSI+GO	0.647	0.969	0.733	0.853	0.949
BRCA	Co-Attention	WSI+GO	0.941	0.969	0.914	0.828	0.962
BRCA	Dual-Attention	WSI+GO	0.682	0.962	0.750	0.901	0.966

Table 6: Comparison of MIL models with different multimodal aggregation mechanisms using WSI+KEGG input based on independent testing. The classification performance of models using only Self-Attention, Co-Attention models, and the proposed MIL model combining Self-Attention and Cross-Attention is reported. Dual-Attention in the table denotes Self-Attention + Cross-Attention (proposed method).

Task	MIL Aggregation	Data	Sensitivity	Specificity	F1-score	PR-AUC	ROC-AUC
LGG	Self-Attention	WSI+KEGG	0.737	0.444	0.651	0.774	0.716
LGG	Co-Attention	WSI+KEGG	0.513	0.778	0.597	0.766	0.721
LGG	Dual-Attention	WSI+KEGG	0.688	0.533	0.647	0.783	0.725
NSCLC	Self-Attention	WSI+KEGG	0.947	0.973	0.960	0.979	0.975
NSCLC	Co-Attention	WSI+KEGG	0.921	0.987	0.952	0.983	0.975
NSCLC	Dual-Attention	WSI+KEGG	0.947	0.903	0.928	0.981	0.982
BRCA	Self-Attention	WSI+KEGG	0.882	0.923	0.811	0.763	0.944
BRCA	Co-Attention	WSI+KEGG	0.882	0.892	0.769	0.760	0.942
BRCA	Dual-Attention	WSI+KEGG	0.682	0.938	0.714	0.843	0.951

Table 7: Backbone comparison of the proposed MIL models with WSI+GO input based on independent testing. The classification performance of the proposed non-end-to-end MIL model using ResNet50d and CTransPath as feature extractors, and the proposed end-to-end MIL model using Reduced MobileNetV4, is reported.

Task	Backbone	Data	Sensitivity	Specificity	F1-score	PR-AUC	ROC-AUC
LGG	ResNet50d	WSI+GO	0.667	0.667	0.675	0.735	0.721
LGG	CTransPath	WSI+GO	0.615	0.694	0.649	0.765	0.716
LGG	MobileNetV4	WSI+GO	0.688	0.578	0.660	0.779	0.740
NSCLC	ResNet50d	WSI+GO	0.882	0.838	0.865	0.939	0.946
NSCLC	CTransPath	WSI+GO	0.855	0.933	0.890	0.972	0.969
NSCLC	MobileNetV4	WSI+GO	0.947	0.935	0.942	0.969	0.978
BRCA	ResNet50d	WSI+GO	0.722	0.922	0.722	0.800	0.939
BRCA	CTransPath	WSI+GO	0.824	0.923	0.778	0.764	0.937
BRCA	MobileNetV4	WSI+GO	0.682	0.962	0.750	0.901	0.966

Table 8: Backbone comparison of the proposed MIL models with WSI+KEGG input based on independent testing. The classification performance of the proposed non-end-to-end MIL model using ResNet50d and CTransPath as feature extractors, and the proposed end-to-end MIL model using Reduced MobileNetV4, is reported.

Task	Backbone	Data	Sensitivity	Specificity	F1-score	PR-AUC	ROC-AUC
LGG	ResNet50d	WSI+KEGG	0.667	0.694	0.684	0.716	0.721
LGG	CTransPath	WSI+KEGG	0.641	0.611	0.641	0.758	0.703
LGG	MobileNetV4	WSI+KEGG	0.688	0.533	0.647	0.783	0.725
NSCLC	ResNet50d	WSI+KEGG	0.895	0.960	0.925	0.979	0.979
NSCLC	CTransPath	WSI+KEGG	0.895	0.920	0.907	0.979	0.971
NSCLC	MobileNetV4	WSI+KEGG	0.947	0.903	0.928	0.981	0.982
BRCA	ResNet50d	WSI+KEGG	0.294	0.969	0.417	0.705	0.911
BRCA	CTransPath	WSI+KEGG	0.500	0.938	0.581	0.732	0.909
BRCA	MobileNetV4	WSI+KEGG	0.682	0.938	0.714	0.843	0.951

Table 9: Comparison of classification performance with different gene representation schemes based on independent testing. The classification performance of the end-to-end MIL model using WSI and individual gene vectors (WSI+individual gene), WSI and GO (WSI+GO), and WSI and KEGG (WSI+KEGG) as inputs is reported.

Task	Data	Sensitivity	Specificity	F1-score	PR-AUC	ROC-AUC
LGG	WSI+Individual gene	0.646	0.644	0.653	0.773	0.719
LGG	WSI+GO	0.688	0.578	0.66	0.779	0.740
LGG	WSI+KEGG	0.688	0.533	0.647	0.783	0.725
NSCLC	WSI+Individual gene	0.895	0.914	0.904	0.964	0.966
NSCLC	WSI+GO	0.947	0.935	0.942	0.969	0.978
NSCLC	WSI+KEGG	0.947	0.903	0.928	0.981	0.982
BRCA	WSI+Individual gene	0.591	0.938	0.650	0.752	0.923
BRCA	WSI+GO	0.682	0.962	0.750	0.901	0.966
BRCA	WSI+KEGG	0.682	0.938	0.714	0.843	0.951
